# Supplementary material for: Validating the Use of Gaussian Process Regression for Adaptive Mapping of Residual Stress Fields
Source: Materials (Basel). 2023 May 20;16(10):3854. doi: 10.3390/ma16103854 (PMC10224383; doi:10.3390/ma16103854)
Supplement: Supplementary file 1 [file materials-16-03854-s001.zip › materials-2321452-supplementary.pdf]

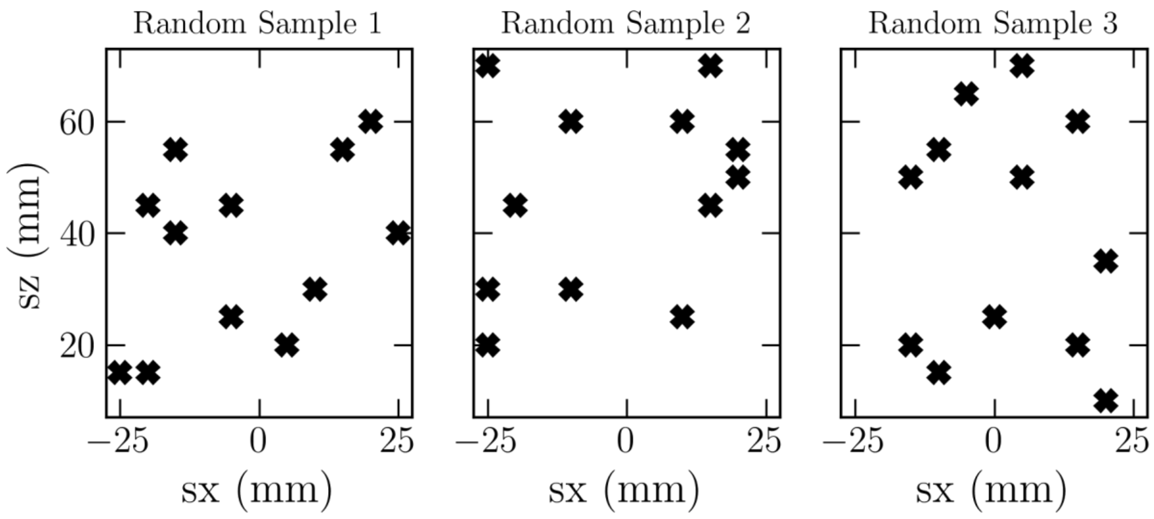

Figure S1. Example locations used to initialize GPRs using a random grid sample.

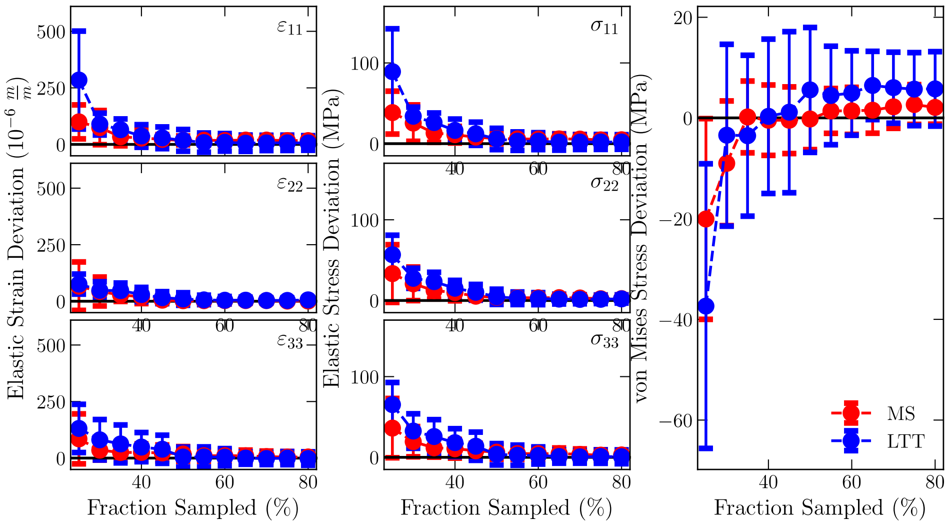

Figure S2. Comparison of how deviations in the estimated interatomic spacing of masked points propagate to errors into strain and, subsequently, stress. Data represent the average deviation between the reconstructed and measured strains and stresses.

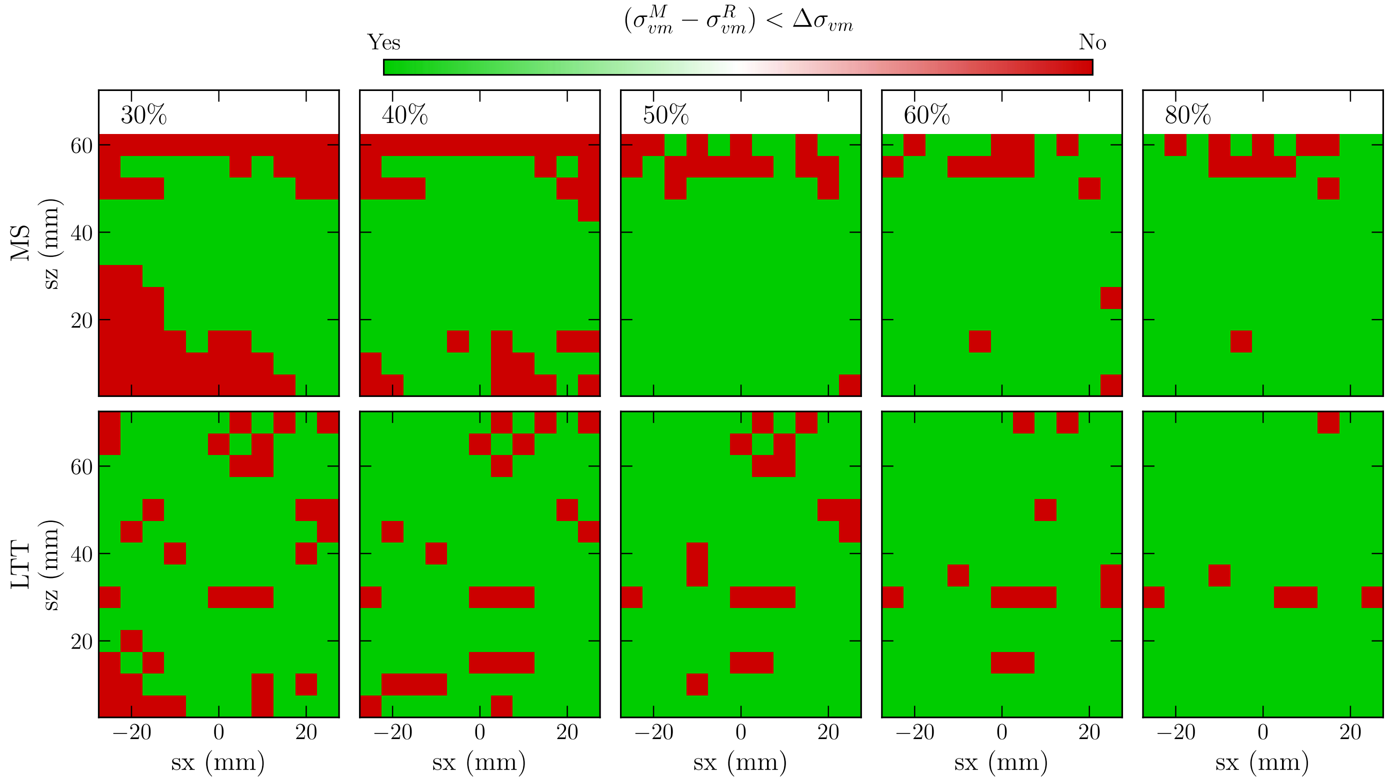

Figure S3. Comparison of the evolution in number of locations where the difference between the measured and reconstructed von Mises stresses exceed the experimental uncertainty. .
